# Therapeutic potential of TRPM8 channels in cancer treatment

**DOI:** 10.3389/fphar.2023.1098448

**Published:** 2023-03-22

**Authors:** Sara V. Ochoa, Zulma Casas, Sonia L. Albarracín, Jhon Jairo Sutachan, Yolima P. Torres

**Affiliations:** ^1^ Departamento de Nutrición y Bioquímica, Pontificia Universidad Javeriana, Bogotá, Colombia; ^2^ Semillero de Investigación, Biofísica y Fisiología de Canales Iónicos, Pontificia Universidad Javeriana, Bogotá, Colombia

**Keywords:** TRPM8, cancer, molecular target, signaling pathway, TRPM8 pharmacology

## Abstract

Cancer is a multifactorial process associated with changes in signaling pathways leading to cell cycle variations and gene expression. The transient receptor potential melastatin 8 (TRPM8) channel is a non-selective cation channel expressed in neuronal and non-neuronal tissues, where it is involved in several processes, including thermosensation, differentiation, and migration. Cancer is a multifactorial process associated with changes in signaling pathways leading to variations in cell cycle and gene expression. Interestingly, it has been shown that TRPM8 channels also participate in physiological processes related to cancer, such as proliferation, survival, and invasion. For instance, TRPM8 channels have an important role in the diagnosis, prognosis, and treatment of prostate cancer. In addition, it has been reported that TRPM8 channels are involved in the progress of pancreatic, breast, bladder, colon, gastric, and skin cancers, glioblastoma, and neuroblastoma. In this review, we summarize the current knowledge on the role of TRPM8 channels in cancer progression. We also discuss the therapeutic potential of TRPM8 in carcinogenesis, which has been proposed as a molecular target for cancer therapy.

## Introduction

Cancer is a non-communicable disease described as one of the most important global health problems and a leading cause of death before the age of 70 in 91 of 172 countries (Bray et al., 2018; Siegel et al., 2021). Chemotherapy and radiotherapy are the most effective options for treating diverse cancers (Dinakar et al., 2022). However, therapy resistance observed in some cancers or stages is responsible for therapeutic failure, metastasis, recurrence, and bad prognosis in patients (Klumpp et al., 2017; Nomura et al., 2021; Hashemi et al., 2022). Research in new and more effective therapies is being developed to improve cancer treatment’s efficacy. There are reports about new delivery technologies (Grolez et al., 2019b; Dinakar et al., 2022), combination therapy (Mokhtari et al., 2017; Liu T. et al., 2018), and the use of diverse proteins as therapeutic targets. Ion channel activity and associated signaling pathways are highly regulated in cancer, and TRPM8 has been proposed as a molecular target in cancer development and progression (Yee, 2015; Liu et al., 2016; Hantute-Ghesquier et al., 2018; Liu et al., 2020). TRPM8 is a non-selective cation channel with a preference for Ca^2+^ (McKemy et al., 2002; Bautista et al., 2007). Channel activity is related to diverse physiological processes, including proliferation and migration, and various reports have proposed TRPM8 channels as therapeutic targets for different cancer treatments, including bone, breast, skin, bladder, pancreas, prostate lung, and colon, among others (Borrelli et al., 2014; Ceylan et al., 2016; Liu J. F. et al., 2018; Du et al., 2018; Lan et al., 2019; Liu J. J. et al., 2022; Koivisto et al., 2022). This review aims to describe the role of the TRPM8 channel in carcinogenesis and the therapeutic potential of this channel in cancer treatment. Furthermore, it is given a current perspective of TRPM8 regulating molecules that could be used in cancer therapy alone or combined with other known drugs or therapies.

## Structure and modulation of TRPM8 channels

Transient receptor potential (TRP) channels are a family of non-selective cation channels formed by different members with diverse biophysical and pharmacological properties. The melastatin-subfamily member 8 (TRPM8) channel is permeable to monovalent and divalent cations, including Na^+^, K^+^, and Ca^2+^, with higher permeability for Ca^2+^ (P_Ca_/P_Na_ = 3.2; McKemy et al., 2002). The channel can be activated by low temperature (<25 °C) and cooling agents such as menthol and icilin, in addition to membrane depolarization (McKemy et al., 2002; Bautista et al., 2007; Yee, 2016). TRPM8 channels are tetrameric proteins with six transmembrane domains (S1-S6) and intracellular N-terminal and C-terminal. The S2-S4 region has a high-affinity menthol binding region (Janssens and Voets, 2011), and the S3 domain has specific amino acids for icilin sensitivity (Chuang et al., 2004). The S4 segment, together with the S4-S5 linker, contains the voltage sensor (Voets et al., 2007a), and transmembrane domains S5 and S6 form the channel pore (Voets et al., 2007b). The intracellular C-terminal has a coiled-coil domain necessary for channel tetramerization (Tsuruda et al., 2006). In addition, it also has a region called the TRP domain which is essential for the channel regulation by temperature and phosphatidylinositol 4,5-biphosphate (PIP2; Liu and Qin, 2005; Rohács et al., 2005; Bandell et al., 2006; Phelps and Gaudet, 2007). TRPM8 phosphorylation in the N-terminus domain alters gating properties that negatively modulate the channel activity and modify the channel response to agonists (Rivera et al., 2021).

Diverse endogenous molecules modulate TRPM8 channel activity and expression. For example, PIP2 is necessary for TRPM8 activation induced by cold and menthol (Rohács et al., 2005; Zakharian et al., 2010). TRPM8 channel activity can also be controlled by TRPM8 channel-associated factors 1 and 2 (TCAF1 and TCAF2), which are regulatory proteins that bind to the channel and modulate their activity. The effects of both proteins are opposite as TCAF1 promotes the channel opening while TCAF2 restrains the channel activity (Gkika et al., 2015). Testosterone and estradiol have been reported to bind TRPM8 channels and modulate channel activity or expression. Treatment of human prostate cells with testosterone promotes a Ca^2+^ current that was inhibited by a TRPM8 channel antagonist, suggesting that the channel is a testosterone receptor (Asuthkar et al., 2015a). Estradiol has been demonstrated to regulate TRPM8 channel expression in breast cancer cell lines (Chodon et al., 2010). (Wondergem et al., 2008). Similarly, nerve growth factor (NGF) induced functional upregulation of the channel through increased channel expression (Kayama et al., 2017). The endocannabinoid anandamide has been shown to have an endogenous functional antagonist effect on TRPM8 channels as anandamide treatment promotes a decreased Ca^2+^ influx induced by icilin and menthol (De Petrocellis et al., 2007). Furthermore, extracellular and intracellular pH changes can modify TRPM8 channel activation properties, reducing the current amplitude and shifting the threshold for channel activation (Andersson et al., 2004). That is interesting, considering the reported changes in pH in the tumoral microenvironmental (Kato et al., 2013), which could regulate the TRPM8 channel activity.

TRPM8 channels are expressed in neuronal and non-neuronal tissues, including the breast, pancreas, bone, and colon, where they are involved in several physiological processes, including cell proliferation and differentiation. In some of these tissues, channel overexpression has been associated with events characteristic of tumors, like cell migration and invasion. However, in some cancers, it has been reported that TRPM8 channel activation is enough to promote proliferation and migration in some tissues but decrease these processes in others (Liu J. F. et al., 2018; Lan et al., 2019; Liu et al., 2020; Henao et al., 2021; Liu J. J. et al., 2022). In the next section, we will describe the reported functions of TRPM8 in diverse cancers.

## Role of TRPM8 channels in cancer

Different reports demonstrate the role of TRPM8 channels in cancer through the modulation of channel expression or activity. TRPM8 is expressed in diverse cancer cells lines and tissues from patients with cancer, including colon cancer (Tsavaler et al., 2001; Borrelli et al., 2014; Liu J. J. et al., 2022); gastric cancer (Xu et al., 2021), oral squamous carcinoma (Okamoto et al., 2012), esophageal carcinoma (Lan et al., 2019), lung cancer (Du et al., 2014; Lai et al., 2020), osteosarcoma (Wang et al., 2013; Zhao and Xu, 2016; Liu Y. et al., 2022), breast cancer (Liu J. et al., 2014), skin cancer (Lan et al., 2019), glioblastoma (Alptekin et al., 2015; Zeng et al., 2019), pancreatic cancer (Liu J. F. et al., 2018) and prostate cancer (Bidaux et al., 2007), among others (Table 1). In addition, overexpression of TRPM8 channels has been demonstrated in gastric, colon, and bone cancers, with a higher expression in patients with metastases. In breast cancer, TRPM8 expression is hormone dependent as steroid deficiency induced a decrease in TRPM8 mRNA. This effect can be counteracted by the treatment with 17β-Estradiol, which restores TRPM8 mRNA levels decreased by steroid deprivation. In addition, silencing estrogen receptors (ERα) promotes a reduction in mRNA TRPM8 expression, suggesting that the channel expression is regulated by ERa (Chodon et al., 2010). Some reports demonstrated that TRPM8 is significantly overexpressed in the cell membrane and cytoplasm of human bladder cancer cells (Li et al., 2010; Wang et al., 2020). However, a considerable reduction in the expression level of TRPM8 mRNA was also reported in bladder cancer tissues. The authors suggest that observed differences could be related to variations in tumor stage (Ceylan et al., 2016; Table 1).

**TABLE 1:** Expression of TRPM8 in diverse cell cancer lines and tissues from patients.

Cancer	TRPM8 expression	Assay	References
***Colon cancer*** Cell lines SW480; CT26 Cell lines Caco-2, HCT116 Tissues from patients with colon cancer	Upregulated in cell lines and tissues	RT-qPCR Western blot	Jia-Jun Liu et al. (2022), Tsavaler et al. (2001), Borrelli et al. (2014)
***Bone cancer*** Cell lines U2OS, MG-63, SaOS2, HOS, 143b. Tissues from patients with colon cancer	Overexpression in cell lines and tissues	RT-qPCR, Western blot, immunohistochemistry	Wang et al. (2013), Zhao and Xu (2016), Xu et al. (2021), Yujie Liu et al. (2022)
***Gastric cancer*** Cell lines SNU-1, AGS, SNU-5, NCI-N87. Human gastric cell line HGG-27	Overexpression in cell lines and tissues	Western blot, immunohistochemistry; RT-qPCR Western blot	Lan et al. (2019), Xu et al. (2021)
***Oral cancer*** Cell lines HSC3 and HSC4	High expression	RT-qPCR, immunofluorescence, patch clamp	Okamoto et al. (2012)
***Esophagus cancer*** Human esophageal cancer cell lines: EC109, KYSE-150, TE-1, TE-10. Tissues from patients with esophageal cancer	Upregulated expression	RT-qPCR Western blot	Lan et al. (2019)
***Lung cancer*** Human lung cancer cell line A549 Lewis Lun Cancer	Upregulated expression	RT-qPCR Western blot	Du et al. (2014), Lan et al. (2019)
***Breast cancer*** Human breast cancer cell line MDA-MB-231. Human breast cancer cell line MCF-7. Human breast cancer cell line MCF-7; Human breast cancer epithelial primary culture (hBCE). Human breast cancer cell lines MCF-7, T47D, MDA-MB-231, BT549, SKBR3, ZR-75-30	Upregulated expression. High expression. High expression	RT-qPCR Western blot. RT-qPCR Western blot, immunohistochemistry. RT-qPCR, immunohistochemistry. RT-qPCR Western blot	Chodon et al. (2010), Dhennin-Duthille et al. (2011), Jinxin Liu et al. (2014), Lan et al. (2019)
***Skin cancer*** Uveal melanoma cell lines 92.1, Mel 202 and Mel 270. Human malignant melanoma cell line A-375. Tissues from patients with NMSC. B16 melanoma cells Human malignant melanoma cell lines A-375, G361, A2058 Human malignant melanoma cell line G361	Overexpression in uveal melanoma cell lines. Non-expression in human healthy uveal tissue. High expression Higher expression than SCC (squamous cell carcinoma). Expression Overexpression in A-375 and A2058. Expression	RT-qPCR, immunohistochemistry, patch clamp. RT-PCR Immunohistochemistry Immunofluorescence RT-PCR Western blot. RT-PCR, immunohistochemistry, patch clamp, Cell-based *in situ* hybridization	Lan et al. (2019), Mergler et al. (2014), Kijpornyongpan et al. (2014); Hemida et al. (2021); Nomura et al. (2021), Zheng et al. (2019); Yamamura et al. (2008)
***Blader cancer*** Human bladder cancer cells T24. Tissue from patients with urothelial carcinoma of bladder. Tissues from patients with bladder cancer. Human bladder cancer lines T24 and EJ	Highly expressed. Overexpressed. Reduction in the expression level. Highly expressed	RT-qPCR, Western blot, immunohistochemistry. RT-qPCR, immunohistochemistry. RT-qPCR, immunohistochemistry. RT-qPCR Western blot	Li et al. (2010), Xiao et al. (2014), Ceylan et al. (2016)
***Pancreatic cancer*** Human pancreatic adenocarcinoma cell lines BxPC-3, Capan-1, HPAF-II, MIA PaCa-2, PANC-1, Panc 02.30, PL45. Human pancreatic adenocarcinoma tissues. Pancreatic ductal adenocarcinoma cell lines Panc-1, PK-9, MiaPaCa-2. Primary pancreatic cancer tissues. Human pancreatic cancer cell lines SW 1990, PANC-1, CAPAN-1, BXPC3. Tissue from patients with pancreatic cancer	Overexpressed. Expressed. Increased expression. Increased expression	RT-qPCR, Immunohistochemistry, immunocytochemistry. Western blot, Immunocytochemistry. RT-qPCR, Western blot, immunohistochemistry. RT-qPCR.	Yee et al. (2010), Cucu et al. (2014), Bin Liu et al. (2018), Du et al. (2018)
***Prostate cancer*** Cell lines LNCaP, DU145 and PC3. Cell lines LNCaP, DU145, PC3 and BPH-1; Tissues from patients with prostate cancer	Channel overexpression. Channel overexpression	RT-qPCR RT-qPCR	Schmidt et al. (2006), Valero et al. (2012)
***Glioblastoma*** Human glioblastoma multiforme cell, DBTRG. Human glioblastoma cell line T98G, U-87MG, U-251	Channel expression. High expression	RT-qPCR Immunoblot. RT-qPCR, Immunoblot, patch clamp	Wondergem et al. (2008), Klumpp et al. (2017)

TRPM8 channel activity is also involved in cancer development and progression, and channel overexpression is regularly associated with poor prognosis. For example, in gastric, pancreatic, and bone cancers, adenocarcinoma, and glioblastoma, TRPM8 promotes invasion and metastasis. Moreover, the high expression has been correlated with a stronger aggressiveness, larger tumor size and clinical stage, shorter overall survival rate, and poor prognosis (Dhennin-Duthille et al., 2011; Yee et al., 2012b; Liu J. et al., 2014; Yee et al., 2014; Alptekin et al., 2015; Zhao and Xu, 2016; Liu J. F. et al., 2018; Du et al., 2018; Patel et al., 2019; Zeng et al., 2019; Xu et al., 2021; Liu Y. et al., 2022). Similarly, tissues from patients with urothelial carcinoma of the bladder showed an association of the channel expression with histologic grade and tumor stage, as patients with high TRPM8 expression showed a shorter overall survival and a poor outcome compared with patients with low channel expression (Xiao et al., 2014). In addition, in squamous cell carcinoma (SCC) non-melanoma skin cancer (NMSC), a higher expression of the channel was correlated with tumor size and poor tumor differentiation (Hemida et al., 2021).

**FIGURE 1:**
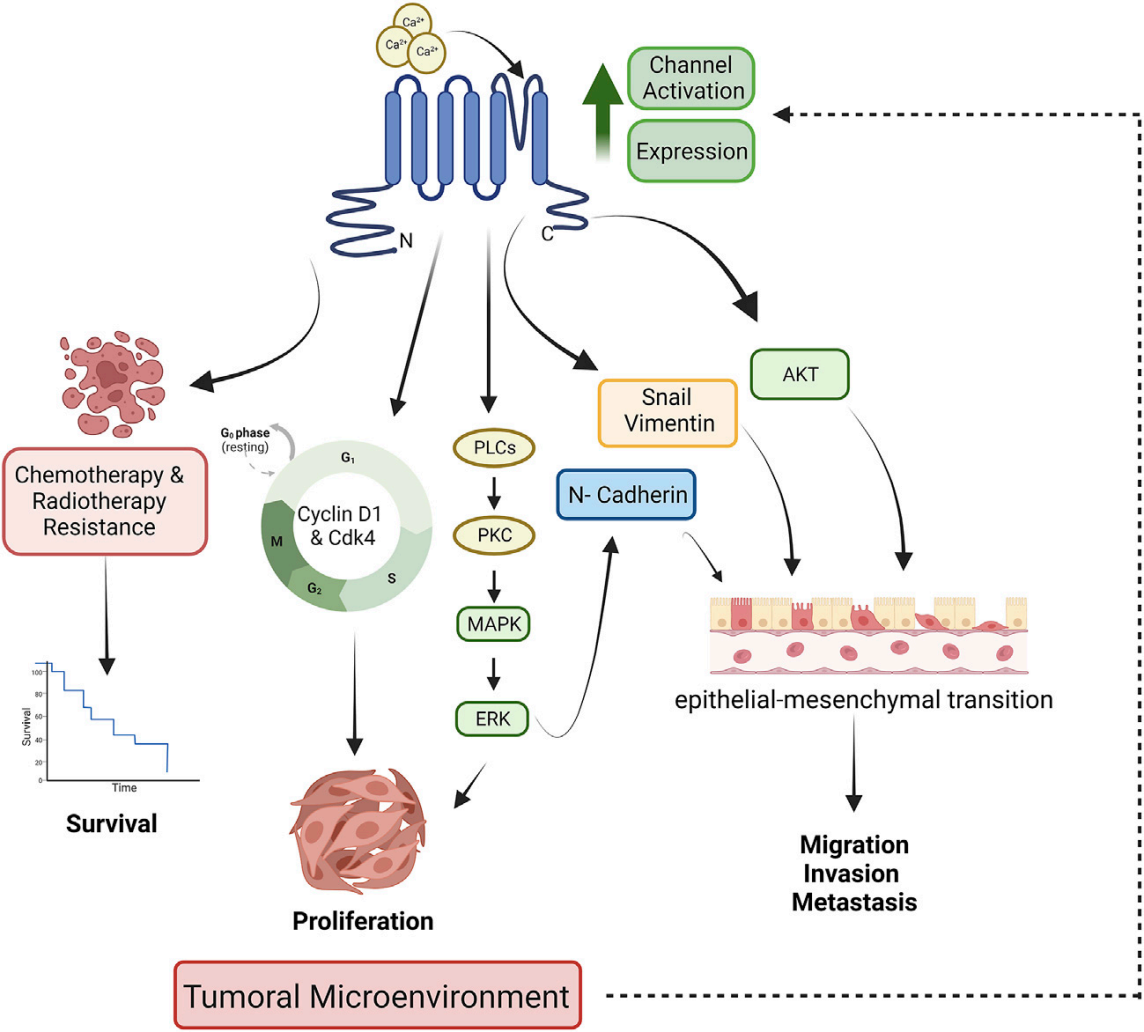
TRPM8-dependent mechanisms involved in tumor development. The tumoral microenvironment releases factors that increase the expression and activation of TRPM8 channels. Channel activation leads to the activation of signaling pathways whose activation decreases patient survival and increases proliferation, migration, invasiveness, and metastasis. Designed with biorender.

The effects of TRPM8 channels in cancer prognosis are associated with modulation of cell viability, proliferation, migration, and apoptosis; phenomena demonstrated employing TRPM8 overexpression, knockdown, and channel regulation activity by agonist or antagonist (Figure 1). For instance, the upregulation of TRPM8 in colon cancer cells has been related to higher proliferation, migration, invasion, and metastasis of tumor cells. In contrast, channel inhibition reduces cell viability of colorectal carcinoma cell lines Caco-2 and HCT116 and promotes apoptosis (Tsavaler et al., 2001; Borrelli et al., 2014; Liu J. J. et al., 2022; Politi et al., 2022). A similar effect on cell proliferation was observed in gastric cancer (Xu et al., 2021). In oral squamous cell carcinoma (SCC), proliferation was not affected by channel activity; however, an important effect of TRPM8 in cell migration was observed, which was abolished by channel inhibition with antagonists. Interestingly, the cell migration level was lower than observed in control cells, suggesting that basally activated channels are involved in SCC migration (Okamoto et al., 2012). Esophageal cancer is another malignant tumor of the digestive system with a high incidence and mortality (Bray et al., 2018); in this kind of tumor, TRPM8 channels have an important effect in promoting cell viability and avoiding apoptosis (Lan et al., 2019). In bone cancer, pancreatic adenocarcinoma, glioblastoma, and bladder cancer, it was also demonstrated that TRPM8 promotes cell proliferation and migration. At the same time, its inhibition induces cell apoptosis (Yee et al., 2010; Wang et al., 2013; Alptekin et al., 2015; Liu J. F. et al., 2018; Zeng et al., 2019; Wang et al., 2020).

The effect of the channel on the growth, death, and apoptosis of bladder cancer cells was regulated by mitochondrial activity (Li et al., 2010; Xiao et al., 2014). It has been described that in glioblastoma, TRPM8 channels modulate the expression of apoptosis-related factors through regulation of the p38/MAPK (mitogen-activated protein kinases) pathway (Alptekin et al., 2015; Zeng et al., 2019). By using Lewis lung cancer (LLC), with different expression ratios of TRPM8 and TRPA1, it was observed that cells with a similar expression of TRPM8 and TRPA1 channels had less autophagy, greater migration, and greater invasiveness than cells with higher or lower TRPM8 expression. The previous results suggest that expression levels of TRPM8 are relevant in lung cancer (Du et al., 2014). Moreover, autophagy mediates breast cancer proliferation and migration by regulating TRPM8 expression and activity. TRPM8 overexpression and activation promote autophagy, while downregulation of the channel decreases it (Huang et al., 2020). In addition, wound healing and transwell assays demonstrated that high expression of TRPM8 promotes a fast movement and invasive ability in breast cancer cells (Liu J. et al., 2014).

In prostate cancer (PCa) cells, the expression of the TRPM8 channel is controlled by androgens *via* androgen receptor (AR) activation. This regulation occurs in lipid rafts microdomains where TRPM8 channels interact with the AR in a testosterone-dependent way, leading to translocation from the endoplasmic reticulum to the plasma membrane (Henshall et al., 2003; Bidaux et al., 2007). At initial PCa stages, the channel has a strong expression, which is lost in late and more aggressive advanced stages (Schmidt et al., 2006; Lunardi et al., 2021). The role of TRPM8 channels in prostate cancer is still controversial due to reports showing both pro-proliferative/antiproliferative and pro-apoptotic/anti-apoptotic effects of the channel. It was proposed that this discrepancy is related to differences in the androgen-sensitivity of cells as TRPM8 promotes proliferation in PCa androgen-sensitive cells LNCaP, but decreases proliferation in androgen-insensitive cells PC3 and DU145 (Yang et al., 2009; Zhu et al., 2011; Wang et al., 2012; Asuthkar et al., 2015b; Grolez and Gkika, 2016). However, channel inhibition or knockdown decreases proliferation in LNCaP, DU145, and PC3 cells, demonstrating a pro-proliferation role of TRPM8 channels independently of androgen-sensitivity (Valero et al., 2012; Liu T. et al., 2018). Anti-apoptotic role of the channel was observed in androgen-dependent LNCaP cells where channel activation promotes the expression of p53 and Caspase-9. On the other hand, a pro-apoptotic effect was demonstrated in both androgen-dependent and androgen-independent cells (Zhang and Barritt, 2004; Wang et al., 2012; Peng et al., 2015). Dissimilar results suggest that additional factors modulate the cell response to TRPM8 channel activity. The tumoral microenvironment has been proposed to modulate diverse cell processes, including ion channel activity (Pethő et al., 2019). It is feasible to consider that it could affect the channel response in prostate cancer.

TRPM8 channels modulate molecules involved in diverse mechanisms and pathways signaling related to the regulation of cell cycle progression, motility, and invasiveness, among others (Adiga et al., 2022). In addition to Ca^2+^, TRPM8 opening trigger the influx of Na^+^ and K^+^ ions which are essential for the control of diverse process involved in cancer progression as proliferation, cell volume regulation and apoptosis (Zhang and Barritt, 2006; Huang and January 2014). However, considering the higher permeability of Ca^2+^ ions, most of the literature about the role of the TRPM8 channel in cancer is focused on Ca^2+^ signaling (McKemy et al., 2002). In osteosarcoma and glioblastoma, TRPM8 promotes cell proliferation through the modulation of cyclin D1 and cyclin-dependent kinase 4 (Cdk4) expression (Wang et al., 2013; Alptekin et al., 2015; Zeng et al., 2019). In pancreatic tissue and diverse cell lines, it was demonstrated that TRPM8 overexpression has a role in cell proliferation by upregulating cyclin-dependent kinase inhibitors expression and promoting cell cycle progression (Yee et al., 2010). Knockdown of TRPM8 induced senescence and a significant decrease in cell proliferation. It was also observed that an increased proportion of cells in the G0/G1 phases and a decrease in the S and G2/M phases (Yee et al., 2010; Yee et al., 2012a; Liu J. F. et al., 2018). In addition, Yee et al. (2012a), Yee et al. (2012b) suggested that TRPM8 activation promotes an increase in intracellular Ca^2+^ concentration, leading to the activation of phospholipase C (PLC) and protein kinase C (PKC), which in turn activate the RAS/ERK pathway and regulate the transcription of diverse genes involved in cellular proliferation and survival (Yee et al., 2012a). The effect of the TRPM8 channel in PCa cells proliferation is related to the modulation of Cdk4 and Cdk6 expression (Yang et al., 2009; Yang et al., 2009; Wang et al., 2012; Asuthkar et al., 2015b; Gkika et al., 2015; Grolez et al., 2019a; Chinigò et al., 2022; Grolez et al., 2022).

In the colon, oral, esophageal, bladder, and breast cancers, have been described that TRPM8 is directly involved in invasiveness and metastasis by regulation of epithelial-mesenchymal transition (EMT; Liu J. J. et al., 2022; Chen et al., 2017; Wang et al., 2020; Liu J. et al., 2014). EMT is a process where epithelial cells lose their apical polarity and adopt a mesenchymal phenotype, thereby increasing properties such as migration and invasiveness as well as resistance to apoptosis, which are directly involved in cancer metastasis. This process involves changes in the expression of mesenchymal markers, including the calcium-dependent membrane proteins N-cadherin and E-cadherin, as well as vimentin and snail, among others (Kaufhold and Bonavida, 2014; Liu et al., 2015). E-cadherin participates in preserving the epithelial phenotype. In contrast, N-cadherin is prevalent in non-epithelial tissues, where it facilitates the activation of MAPK/extracellular signal-regulated kinases (ERK) and phosphoinositide-3-kinase (PI3K) pathways to enhance cell survival and migration in non-epithelial cells (Mrozik et al., 2018; Loh et al., 2019). In addition, proteins involved in cytoskeleton remodeling and cell adhesion, such as vimentin and snail, are also upregulated in EMT. Upregulation of N-cadherin, vimentin, and snail, along with the downregulation of E-cadherin, promotes cancer development by increasing cell migration and metastasis (Kaufhold and Bonavida, 2014; Loh et al., 2019). By transfecting siRNA-TRPM8 in SW480 (human colon cancer cell line) and CT26 (mouse colorectal adenocarcinoma cell line) and tissues samples from patients with colon cancer and liver metastasis, it was observed that TRPM8 upregulates vimentin, snail an N-cadherin while decreasing the expression of E-cadherin. Moreover, silencing of TRPM8 leads to the inhibition of EMT and decreases the proliferation, migration, invasion, and metastasis of colon cancer cells, proving the importance of the channel in these processes (Liu J. J. et al., 2022). Changes in vimentin and snail expression were also reported in bladder cancer in addition to the increase in the expression of b-catenin and paxillin, which are involved in the control of EMT markers and cell adhesion, respectively (Tumbarello et al., 2005; Kim et al., 2019; Wen et al., 2020).

AKT/GSK3-β pathway has an important role in the regulation of the mesenchymal markers (vimentin, snail, and E-cadherin) and the EMT process (Chen et al., 2013; Liu L. et al., 2014). GSK3-β participates in the degradation of some proteins, including snail1 and smad, which inhibit activation of EMT, being critical for tumorigenicity (Gonzalez and Medici, 2014; Nishimura et al., 2016). Activation of AKT signaling inhibits GSK-3β by phosphorylation and promotes EMT (Zhou et al., 2004; Bachelder et al., 2005; Kitagishi et al., 2012; Gonzalez and Medici, 2014; Loh et al., 2019). In bladder, breast, and bone cancer, it was reported that TRPM8 modulates AKT/GSK-3β signaling (Wang et al., 2013; Liu J. et al., 2014; Wang et al., 2020). The proposed mechanism suggests that TRPM8 promotes AKT phosphorylation which decreases GSK-3β and increases snail and vimentin expression and E-cadherin degradation (Liu J. et al., 2014). Knockdown of TRPM8 in bladder cancer cells decreases p-AKT and increases p-GSK-3β level, which alters the process regulated by the AKT/GSK-3β signaling (Wang et al., 2020).

Expression of membrane matrix metalloproteinases (MMPs) is increased in EMT to mediate the remodeling and degradation of extracellular matrix and promote cell invasion and metastasis (Kessenbrock et al., 2010; Pang et al., 2016). MMPs are overexpressed in the late stage of some cancer like head and neck SCC (Antra et al., 2022; Chan et al., 2022). In oral, bone, and prostate cancer, TRPM8 upregulates the activity of MMP2 and MMP9, stimulating invasiveness (Okamoto et al., 2012; Wang et al., 2013). Furthermore, MMPs can degrade E-cadherin and promote EMT (Gonzalez and Medici, 2014). The extracellular-signal-regulated kinase (ERK) one and two are proteins that belong to the family of MAPK and contribute to EMT (Olea-Flores et al., 2019). In diverse cancers, levels of p-ERK1/2 were correlated with upregulation of vimentin, snail, N-cadherin, and MMP-9, with a decrease in E-cadherin expression (Hong et al., 2016; Liu B. et al., 2018). In bone, bladder, prostate cancer, and glioblastoma, TRPM8 regulates the expression of ERK1/2. Knockdown of the channel decreased ERK phosphorylation together with migration and invasion (Wang et al., 2013; Alptekin et al., 2015; Liu T. et al., 2018; Zeng et al., 2019; Wang et al., 2020).

The programmed cell death one ligand (PD-L1) signaling regulates T cell activation to induce immune evasion and promote EMT in diverse cancers (Datar and Schalper, 2016; Kim et al., 2016; Chen et al., 2017; Raimondi et al., 2017; Aghajani et al., 2020). Upregulation of snail and vimentin and downregulation of E-cadherin is positively correlated with PD-L1 overexpression, suggesting a direct relation between PD-L1 and EMT (Datar and Schalper, 2016). In esophagus cancer, TRPM8 mediates the activation of the calcineurin-NFATc3 signaling pathway, which stimulate the increase in PD-L1 expression (Lan et al., 2019). PD-L1 is significantly associated with esophageal cancer progression as it promotes cell viability, proliferation, migration, and metastasis through the decreasing of anti-tumor response to CD8+T that promotes immune system evasion (Brahmer et al., 2012; Chen et al., 2017; Han et al., 2020). It was also demonstrated that PD-L1 overexpression enhanced the expression of mesenchymal markers related to the EMT phenotype. Furthermore, PD-L1 blockade inhibited tumor progression (Mcdermott and Atkins, 2013; Chen et al., 2017; Lan et al., 2019). PD-L1 has been proposed as a prognostic predictor for human esophageal cancer (Chen et al., 2014; Yagi et al., 2019), and modulating TRPM8 channel activity with antagonist could be a pharmacological strategy to regulate PD-L1 effects in esophageal cells. The described effects of TRPM8 are related to the role of the channel in Ca^2+^ signaling from calcium influx and release from intracellular stores. Changes in the intracellular Ca^2+^ concentration regulate diverse cell processes involved in cancer progression, through the activation or inhibition of cytosolic enzymes that regulate transcription factors, and proteins involved in cell cycle regulation and EMT (Shibasaki et al., 1997; Wang et al., 1999; Kim et al., 2011; Garg et al., 2014; Iamshanova et al., 2017; Adiga et al., 2022).

In pancreatic adenocarcinoma, TRPM8 channels are associated with TRPM8 channel-associated factors 1 and 2 (TCAF1 and TCAF2). TCAF1 and two promote TRPM8 trafficking to the cell surface and modulate the channel activity. The analysis of protein interactions demonstrated that TRPM8 channels are physically connected with TCAF2, which has an inhibitory role on channel activity, promoting prostate cancer cell migration. However, it was not observed that physical interaction with TCAF1 was reported to reduce prostate cancer cell migration (Gkika et al., 2015; Chelaru et al., 2022). TRPM8 and TCAF2 expression levels in pancreatic cancer cells have been associated with cancer invasiveness, metastasis, and tumoral stage (Chelaru et al., 2022).

Modulation of TRPM8 activity by agonists and antagonists has also been associated with the enhanced antitumoral activity of some chemotherapeutics. It was demonstrated that the combination of the channel antagonist AMTB with cisplatin (one of the most used chemotherapeutics in osteosarcoma treatment) has a higher antitumoral effect than cisplatin alone. Treatment with both molecules promotes a significant reduction of tumor size by arresting tumor cell growth and metastasis together with the induction of apoptosis (Liu Y. et al., 2022). In addition, the knockdown of the TRPM8 channel did not affect apoptosis but facilitated the apoptosis induced by the anti-tumor molecule epirubicin (EPI), suggesting a role of the channel in epirubicin resistance (Wang et al., 2013). Furthermore, in lung cancer, the enhanced efficiency of the chemotherapeutic doxorubicin (DOX) was reported in A549 cells treated with the TRPM8 agonist borneol. The proposed mechanism involves the activation of intracellular TRPM8 channels, which trigger intracellular Ca^2+^ mobilization. In addition, the role of TRPM8 channels in apoptosis induced by borneol and DOX was reported, mediated by the overproduction of reactive oxygen species (ROS; Lai et al., 2020). On the contrary, TRPM8 expression decreases pancreatic cancer cell sensitivity to the chemotherapeutic gemcitabine, which is associated with changes in the expression of proteins responsible for multidrug resistance, apoptosis, and gemcitabine metabolism (Liu J. F. et al., 2018).

Radioresistance has been observed after radiation therapy, and TRPM8 contributes to the radioresistance of B16 melanoma cells. TRPM8 expression increases in B16 melanoma cells after g-irradiation, which improves DNA damage response (DDR) for DNA repair and cell recovery. Channel inhibition with the antagonist AMTB or TRPM8 knockdown decreased the tumor growth induced by g-irradiation by suppressing DDR and promoting radio sensitizing. In addition, channel activation with the agonist W-12 increased the survival rate of cancer cells (Nomura et al., 2021). Similar effects were observed in glioblastoma, where TRPM8 participates in cancer cell resistance. Klumpp et al., 2017 reported that ionizing radiation upregulates TRPM8 in glioblastoma and promotes DNA repair and clonogenic survival by cell cycle control and prevention of apoptosis (Klumpp et al., 2017).

Changes in the cell microenvironment observed in cancer progression include modifications in mechanical signals, which can be regulated by ion channels (Butcher et al., 2009; Pethő et al., 2019). In addition, Wei et al. (2015) proved that increased matrix stiffness in the tumor microenvironment directly activates EMT, tumor invasion, and metastasis through the EMT-inducing transcription factor TWIST1 (Wei et al., 2015). TRPM8 has a role in the mechanosensitive response of breast epithelial cells which could be dysregulated by the activation of KRas oncogene. In normal breast epithelial cells, mechanical stimulation induces rapid activation of NADPH-oxidase 2 (NOX2) and the production of ROS. These events promote TRPM8 channel activation and a persistent extracellular calcium influx. Overexpression of the constitutively active KRAs oncogene modifies the Ca^2+^ signaling derived from mechanical stimulation. It reduces the ability of cells to respond to ROS, which could modify cancer cells’ responses to the mechanical microenvironment and affect patient survival (Pratt et al., 2020). In bladder cancer, TRMP8 also regulates ROS production and the expression levels of the enzymes Catalase, HO-1, and SOD2, which regulate tumor growth *in vivo*, demonstrating that TRPM8 has an essential role in bladder cancer (Wang et al., 2020).

Hypoxia is closely associated with cell proliferation, angiogenesis, metabolism, and the tumor immune response (Li et al., 2021). TRPM8 channel overexpression or activation promotes tumor growth under hypoxic conditions in prostate cancer, an effect facilitated by the upregulation of HIF-1α protein through Ca^2+^-dependent inhibition of RACK1(Yu et al., 2014). TRPM8 is preferentially expressed in the plasma membrane in non-cancer cells. However, in tumoral tissues, the channel is removed from the plasma membrane and targeted for degradation. Inhibition of the ubiquitination cascade increases the activity of the TRPM8 channel in the plasma membrane which has been proposed as a therapeutic strategy for regulating cell growth and proliferation in prostate cancer cells (Asuthkar et al., 2015b; Asuthkar et al., 2017).

It was demonstrated that pancreatic cancer cells express a non-glycosylated form of TRPM8 channel with biophysical properties different from other cells. Interestingly, that variant inhibits pancreatic adenocarcinoma cell migration and proliferation, contrary to the reported TRPM8 channel effect, suggesting an important role of post-translational modifications in cancer development (Cucu et al., 2014; Ulăreanu et al., 2017). In addition, Huang et al., 2022 demonstrated the importance of Y1022 phosphorylation in cancer progression. Y1022, ubicated in the C-terminal of TRPM8, is phosphorylated by lymphocyte-specific protein tyrosine kinase (LCK), which modulates channel multimerization and increases the TRPM8 current density. In AsPC-1 and PANC-1 pancreatic cell lines, mutant TRPM8 Y1022F fails to increase cell proliferation and migration, demonstrating the importance of that residue in pancreatic cancer cell proliferation, migration, and tumorigenesis *in vitro* and *in vivo*. In that sense, the authors suggest that channel regulation by LKC may be a potential therapeutic target for pancreatic cancer (Huang et al., 2022).

## Therapeutic potential of TRPM8 in cancer

TRPM8 has been proposed as a therapeutic target for cancer treatment because of its demonstrated role in cancer development and progression. In the last years, diverse molecules have been proven to have *in vitro* and *in vivo* effects on the TRPM8 channel activity. Moreover, some of them have been tested in preclinical and clinical trials (Knowlton and Mckemy, 2011; Liu et al., 2016; Pérez de Vega et al., 2016). This section reviews TRPM8 channel agonists and antagonists, which have been demonstrated to regulate cellular characteristics associated with diverse cancers. We also review information from assays focused on the potential effect of channel modulation in cancer treatment.

TRPM8 agonists modulate various properties in cancer cells, and menthol is one of the most extensively used pharmacological studies (Izquierdo et al., 2021). Menthol is an FDA GRAS (generally regarded as safe) molecule without known considerable adverse effects. In human malignant melanoma cell lines G-361 and A-375, menthol induces a dose-dependent increase in intracellular Ca^2+^ concentration and promotes a cytotoxic effect (Yamamura et al., 2008; Kijpornyongpan et al., 2014). The menthol-induced effect on A-375 cells was comparable to that induced by 5-fluorouracil and was abolished by the antagonist BCTC (Kijpornyongpan et al., 2014). In T24 human bladder cancer cells, menthol increases the intracellular Ca^2+^ concentration, mitochondrial membrane depolarization, and cell death in a dose-dependent way, suggesting the potential use of TRPM8 agonists in the cancer cell (Li et al., 2010). Contrary to these reports, in glioblastoma cells (DBTRG) have been demonstrated that menthol promotes cell migration and stimulates both Ca^2+^ influx and release from intracellular stores in a dose-dependent effect (Wondergem et al., 2008; Wondergem and Bartley, 2009). Similarly, in glioblastoma cell lines U-87MG and T98G were observed that the TRPM8 agonist icilin increased chemotaxis (Klumpp et al., 2017). In both cases, the effect of channel agonists was inhibited by the TRPM8 antagonist 2-Aminoethoxydiphenyl borate (2-APB) and BCTC, respectively (Wondergem et al., 2008; Wondergem and Bartley, 2009; Klumpp et al., 2017). The differential observed effect could be related to the divergent roles of the channel in cancer treatment. A proliferative effect of the channel was observed in some cancers, including breast, pancreas, glioblastoma, and bone cancer; a pro-apoptotic effect was demonstrated in prostate cancer.

Even though the cytotoxic effects induced by menthol in cancer cells, there are no reports from clinical trials using menthol as a therapeutic target in cancer treatment. Considering the role of TRPM8 channels in pain signaling, the clinical trials with menthol have been focused on pain treatment (Fernández-carvajal et al., 2020). For instance, the clinical trial NCT05429814 proposed to evaluate topical menthol in chemotherapy-related peripheral neuropathy in patients with breast cancer. In addition, by applying the TRPM8 agonist in patients with neuropathic pain, Fallon et al., 2015 suggested that topical menthol could potentially treat neuropathic pain derived from chemotherapy (Fallon et al., 2015). Both reports suggest the use of a topical formulation of menthol, which reduces the occurrence of adverse effects. However, it could limit the effectiveness of using these molecules for cancer treatment. Another critical thing restricting the use of menthol as a cancer target is its unselective effect because it has been reported that it can modulate other ion channels (Hansen et al., 2017).

D3263 has been described as a selective TRPM8 agonist. Preclinical studies analyzed the effect of oral administration of D3263 alone or in combination with Finasteride, an FDA-approved pharmacologic agent for treating benign prostate hyperplasia (BPH; Duncan et al., 2009; Tacklind et al., 2010). Results demonstrate a dose-response reduction in mean rat prostate hyperplasia, and an additive effect with Finasteride, suggesting D3263 for BPH treatment (Duncan et al., 2009; Moran et al., 2011). Significantly, in 2009, a Phase I clinical trial (NCT00839631) was carried out to test the effect of D3262 on solid tumors, including prostate, colon, breast, lung, pancreas, leiomyosarcoma, and Kaposi’s sarcoma. After oral administration of D3262 to 23 participants, all patients were reported to feel a cold sensation (like a menthol sensation) on the skin and mucous membranes. Preliminary results of the trial revealed that three men with advanced prostate cancer showed evidence of disease stabilization (Tolcher et al., 2010). Although the study was finished, no clinical results were reported or recent information about the effect of these molecules on cancer progression.

TRPM8 channel antagonist is also proposed as a potential alternative in cancer treatment from results derived from studies with osteosarcoma, bladder, and colorectal cancer among others. Wang et al., 2020 demonstrate that TRPM8 inhibition with BCTC promotes a reduction in cell viability, proliferation, and migration in bladder cancer cells (Wang et al., 2020). Similarly, the TRPM8 antagonist AMTB decreases cell proliferation and migration and promotes apoptosis of osteosarcoma cell lines. The described mechanism suggests that AMTB affects Smad2 and Smad3 phosphorylation and represses the TGFb pathway (Liu Y. et al., 2022). Cannabigerol (CBG), cannabidiol (CBD), and cannabidivarin (CBDV) are cannabinoids with antagonist effects on TRPM8 channels. *In vitro* and *in vivo* assays demonstrated that all of them decrease colorectal cancer cell viability and reduce cell growth (De Petrocellis et al., 2013; Borrelli et al., 2014; Mangal et al., 2021). Borrelli et al. (2014) demonstrated a mechanism associated with apoptosis induction and ROS production in a concentration-dependent way. The TRPM8 antagonist AMTB reproduced a lesser potent effect on cell viability. Furthermore, mRNA TRPM8 silencing decreased the effect of CBG on cell viability (Borrelli et al., 2014). Similarly, a CBD pro-apoptotic effect, p53 activation, and ROS elevation were observed in prostate carcinoma (De Petrocellis et al., 2013). These results lead the authors to suggest the use of non-THC cannabinoids as therapeutic strategies for colorectal cancer and prostate carcinoma (De Petrocellis et al., 2013; Borrelli et al., 2014; Mangal et al., 2021). In esophageal cancer cells, the addition of the TRPM8 antagonist RQ-00203078 decreases cancer proliferation and cell viability by apoptosis induction (Lan et al., 2019). Similarly, oral squamous carcinoma cells treated with the antagonist RQ-00203078 showed a decrease in cell migration. In addition, the antagonist reduced invading ability by suppressing the MMP activity. Considering the importance of invasiveness in oral SCC, channel inhibition could be a potential treatment for that cancer (Okamoto et al., 2012).

There is contradictory information about the effect of TRPM8 modulation in prostate cancer, as both agonists and antagonists have been shown to decrease proliferation and migration and promote apoptosis in PCa cells *in vitro* (Grolez and Gkika, 2016). That effect has been associated with the differences in channel expression during cancer progression. High expression is found in the initial cancer stages, and loss of expression is observed in the late and more aggressive stages (Henshall et al., 2003). In addition, it was demonstrated that the TRPM8 expression level depends on the cell line. Androgen-dependent LNCaP and androgen-independent cells PC-3 and DU145 showed different expression levels of the TRPM8 channel (Henshall et al., 2003).

Recently, using the androgen-independent cell line PC3 with stable heterologous expression of TRPM8 channels (PC3-M8), it was reported that TRPM8 activation limited tumor growth by decreasing clonogenicity and cell proliferation through cell cycle arrest at the G0/G1 phase. Furthermore, by using the agonist WS12 inside lipid nanocapsules, the authors demonstrate that TRPM8 activation decreases cell migration, and metastasis, suggesting a protective role of the channel on prostate cancer progression (Grolez et al., 2019b; Grolez et al., 2022). Invasiveness was also inhibited through the decrease in the EMT by Rac1 and Cdc42 inhibition (Grolez et al., 2022). The effects of TRPM8 inhibition on cell migration demonstrated the nanocarriers’ efficiency in delivering the channel agonist *in vivo*. In addition to the protective effect of TRPM8 in prostate cancer, it was demonstrated the advantage of using nanocarriers on the improved activity of the channel agonist, as encapsulation decreased the concentration required to activate the channel. It is suggested that channel activation with TRPM8 agonist in nanocarriers could be used in the early stages of prostate cancer to decrease tumor cell dissemination (Grolez et al., 2019b; Grolez et al., 2022). In DU145 cells, menthol induced a drop in cell migration and inhibited cell growth without apoptosis. After treatment with menthol, a decrease in cell proliferation and a substantial increase in G0/G1 phase were observed. Furthermore, menthol induces the downregulation of the CDK2, CDK4, and CDK6, involved in cell progression, and phosphor-FAK, which participates in cell migration. These effects were partially abolished by the addition of the TRPM8 channel antagonist BCTC (Wang et al., 2012).

In another way, the positive effect of TRPM8 antagonists for prostate cancer treatment was demonstrated using the androgen-dependent prostate cancer cells LNCaP. Donato et al., 2021 analyzed several molecules and reported that TRPM8 channel antagonists inhibit proliferation, migration, and invasiveness of the androgen-dependent prostate cancer cells LNCaP cells. TRPM8 antagonists reverse the androgen-induced increase in calcium influx and the rapid signaling activation that led to the androgen-elicited invasion and proliferation in various PC-derived cells. Furthermore, LNCaP migration and invasion stimulated by androgens were decreased by channel antagonists (Donato et al., 2021). Sesamin, a molecule from Traditional Chinese medicine, has a selective dose-dependent antagonist effect on the TRPM8 channel. *In vitro* assays demonstrated that Sesamin inhibited cell growth in prostate adenocarcinoma cells LNCaP and DU145 through an antiproliferative effect. However, *in vivo* effects have not been reported (Sui et al., 2020). These results suggest that the effect of channel antagonists or agonists depends on the type of prostate cells, androgen-dependent or androgen-independent. However, it was reported that antagonist AMTB decreased the proliferation and migration of both androgen-dependent and androgen-independent prostate cancer cells, demonstrating that there is an additional mechanism controlling the response of pancreatic cancer cells to TRPM8 channel modulation (Valero et al., 2012).

Combination therapy uses two or more therapeutic agents for cancer treatment (Mokhtari et al., 2017), and there are exciting reports about the potential use of TRPM8 modulators combined with currently used chemotherapeutics. In osteosarcoma, an increased sensitivity to cisplatin was observed when combined with AMTB, suggesting that channel inhibition increase the antitumoral effect of the chemotherapeutic. Similar results were found in a preclinical, experimental model of osteosarcoma (Liu Y. et al., 2022). Following that, knocking down TRPM8 channels in osteosarcoma and prostate cancer cell lines increased apoptosis induced by epirubicin, suggesting an enhancement in chemosensitivity (Wang et al., 2013; Liu T. et al., 2018). In aggressive prostate cancer cells was demonstrated that the agonist D-3263 significantly improves the pro-apoptotic effect of enzalutamide and docetaxel, antiandrogen and antineoplastic, respectively, used in the treatment of prostate cancer (Genovesi et al., 2022).

Furthermore, Nagai et al. (2019) reported that menthol enhances the effect of anti-cancer drugs Paclitaxel and Vincristine on human hepatocellular carcinoma HepG2 cells where cell viability was significantly decreased through downregulation of CYP3A4 (Nagai et al., 2019). Although the reported mechanism is not related to TRPM8 channel activity, it is possible that the increase in intracellular Ca^2+^ concentration, derived from channel activation, led to the modification of the CYP3A4 expression, as has been reported for other cytochromes (Clyne et al., 1996). Radiotherapy is currently used in treating several cancers, and it has been proposed that combining this therapy with TRPM8 modulation could be helpful in cancer therapy. When prostate cancer cells were stimulated with ionizing radiation combined with the TRPM8 agonist WS-12, a significant induction of apoptosis was observed, suggesting that channel activation improves the effect of radiation therapy (Alaimo et al., 2020); Alaimo et al., 2021).

From analyzing the TRPM8 expression, drug activities, and known drug targets, Pan et al., 2022 reported a correlation between the expression of the TRPM8 channel and the response to Paclitaxel (Pan et al., 2022). *Paclitaxel* is a chemotherapeutic used in diverse cancers which acts as a mitotic inhibitor by interfering with the normal function of microtubule growth. Paclitaxel induces an increase in ROS production and stimulates signaling pathways as p38/MAPK, related to apoptosis induction. In addition, it mediates the activation of regulatory proteins Bad and Bax, which promote apoptosis, and Bcl2, which suppresses apoptosis (Kampan et al., 2015; Zhu and Chen, 2019). In human prostate carcinoma, it was demonstrated that TRPM8 channel activation with TRPM8 agonists promotes apoptosis, an effect mediated by an increase in intracellular Ca^2+^ concentration (Zhang and Barritt, 2004; Alaimo et al., 2020).

Furthermore, human prostate carcinoma cells treated with the agonist WS-12 plus docetaxel, an analog of paclitaxel (Pienta, 2001), showed significant activation of apoptotic cell death (Alaimo et al., 2020). Interestingly, paclitaxel promotes a substantial increase in TRPM8 protein expression in IB4(−) neurons, which contributes to heat and cold hypersensitivity observed in chemotherapy-induced peripheral neuropathy (CIPN) induced by paclitaxel. When the channel was inhibited with the antagonist AMTB, the cold-hyperalgesia was reduced (Villalba-Riquelme et al., 2022). Considering these results, it could be interesting to carry on specific assays to evaluate the precise role of TRPM8 in response to paclitaxel in cancer prostate and other cancer cells.

TRPM8 was linked to the effect of other chemotherapeutics, including Lapatinib, Erlotinib, Afatinib, and Dabrafenib, which are approved for treating diverse cancers (Pan et al., 2022; Zhang et al., 2022). FDA approved Afatinib, Dabrafenib, and Erlotinib to treat non-small cell lung cancer (NSCLC; Rosell et al., 2012; Hirsh, 2017; Hu et al., 2017; Yuan et al., 2022). In addition, Afatinib has been proposed for breast and gastric cancer (Lin et al., 2012; Hirsh, 2017; Hu et al., 2017; Zhu et al., 2021); Dabrafenib is reported for the treatment of patients with advanced melanoma and colorectal cancer (Yuan et al., 2022), and Lapatinib is used in the treatment of breast cancer (Segovia-Mendoza et al., 2015; Eustace et al., 2018). Those molecules are tyrosine kinase inhibitors of the epidermal growth factor receptor (EGFR) and ErbB-family that regulate growth inhibition, apoptosis, and autophagy through the modulation of diverse pathways, including PI3K-Akt, the Ras-Raf-MEK-ERK1/2 and the phospholipase C (PLC) pathway (Segovia-Mendoza et al., 2015; Suzawa et al., 2016; Hirsh, 2017; Hu et al., 2017; Sekhon et al., 2017; Khunger et al., 2018; Yuan et al., 2022). ERK1/ERK2 and associated pathways have been reported to be modulated by TRPM8 expression in bone cancer (Wang et al., 2013), bladder cancer (Wang et al., 2020), pancreatic cancer (Yee et al., 2012a) and glioblastoma (Alptekin et al., 2015). In addition, in pancreatic cancer (Yee et al., 2012a) and breast cancer (Pratt et al., 2020), there were shown changes in Ras expression induced by TRPM8 activity. More profound studies are necessary, focusing on the relationship between the TRPM8 channel and the activity of chemotherapeutics currently used in cancer treatment to get an improved therapy using FDA-approved drugs.

There is increasing information about the role of TRPM8 modulation in cancer. However, to our knowledge, there are no TRPM8 modulators FDA-approved for cancer treatment. One important factor that restricts promissory molecules’ selection is channel selectivity. Molecules such as menthol or icilin, two of the most common agonists of TRPM8 channels, and the antagonist BCTC have been demonstrated to activate other members of the TRP family (Hansen et al., 2017; Heber et al., 2020). Another significant consideration is the development of undesired side effects observed in the early phases of clinical trials (Izquierdo et al., 2021). For example, some studies have demonstrated changes in core body temperature regulation and shifts in temperature sensation after using TRPM8 channel modulators (Andrews et al., 2015). In this way, developing drug delivery technology as nanocarriers used by Grolez et al. (2019a), Grolez et al. (2019b) for biodistribution of TRPM8 modulators could improve treatment efficacy and minimize side effects (Din et al., 2017; Grolez et al., 2019b). Finally, an important focus is the possibility of using both TRPM8 agonists and antagonists for cancer treatment (Pérez de Vega et al., 2016). More research is needed to decipher the complex modulatory effect of TRPM8 in the development and progress of diverse cancers, focusing on characterizing molecules highly selective for the TRPM8 channel that could be used in cancer treatment with minimal adverse effects.
